# Low Dose Methotrexate and Vinblastine, Given Weekly to Patients With Desmoid Tumours, is Associated With Major Toxicity

**DOI:** 10.1080/13577140310001644779

**Published:** 2003

**Authors:** René L. van der Hul, Caroline Seynaeve, Bert N. van Geel, Jaap Verweij

**Affiliations:** 1 Department of Surgical Oncology Erasmus Medical Center–Daniel den Hoed Rotterdam The Netherlands; 2 Department of Medical Oncology Erasmus Medical Center–Daniel den Hoed Cancer Center Grone Hilldijk 301 Rotterdam 3075 EA The Netherlands

## Abstract

*Purpose:* To evaluate the tolerance of a low dose chemotherapy regimen for desmoid tumours.

*Patients and methods:* Patients with desmoids for whom radical resection was impossible or related to extensive mutilation were treated with chemotherapy. Treatment consisted of intravenous methotrexate at a dose of 50mg and vinblastine at a dose of 10mg weekly, scheduled to be given for a total period of 1 year. Doses were reduced and/or delayed on an individual basis, depending on the observed type of toxicity.

*Results:* Ten patients (six males; four females), median age 43 years (range17–75), median WHO performance score 1 (range 0–1), were treated. None of them was able to complete the treatment as scheduled, due to observed side effects, while in two patients treatment was also discontinued because of progressive disease. In six patients, less than 50% of the projected administrations and dose could be given. Severe organ toxicity was noted in three patients (one interstitial pneumonitis, two toxic hepatitis), which, however, was reversible in all cases.

*Discussion:* Methotrexate and vinblastine given at this dose and schedule lead to an unacceptable level of toxicity for a long-term treatment, and cannot be recommended for standard use.


*Sarcoma*, September/December 2003, *VOL. 7, NO. 3/4, 153–157*

Taylor & Francis healthsciences

ORIGINAL ARTICLE

### Low dose methotrexate and vinblastine, given weekly to patients with desmoid tumours, is associated with major toxicity

RENÉ L. VAN DER HUL[1], CAROLINE SEYNAEVE[2], BERT N. VAN GEEL[1] & JAAP VERWEIJ[2]

*Departments of [1]Surgical Oncology and [2]Medical Oncology, Erasmus Medical Center – Daniel den Hoed, Rotterdam, The Netherlands*

**Abstract**
*Purpose:* To evaluate the tolerance of a low dose chemotherapy regimen for desmoid tumours.
*Patients and methods:* Patients with desmoids for whom radical resection was impossible or related to extensive mutilation were treated with chemotherapy. Treatment consisted of intravenous methotrexate at a dose of 50 mg and vinblastine at a dose of 10 mg weekly, scheduled to be given for a total period of 1 year. Doses were reduced and/or delayed on an individual basis, depending on the observed type of toxicity.
*Results:* Ten patients (six males; four females), median age 43 years (range17–75), median WHO performance score 1 (range 0–1), were treated. None of them was able to complete the treatment as scheduled, due to observed side effects, while in two patients treatment was also discontinued because of progressive disease. In six patients, less than 50% of the projected administrations and dose could be given. Severe organ toxicity was noted in three patients (one interstitial pneumonitis, two toxic hepatitis), which, however, was reversible in all cases.
*Discussion:* Methotrexate and vinblastine given at this dose and schedule lead to an unacceptable level of toxicity for a long-term treatment, and cannot be recommended for standard use.

**Key words:** *desmoids, methotrexate, vinblastine, sarcomas*

### Introduction

Desmoid tumours arise from musculoaponeurotic tissues and aggressively infiltrate along fascial planes. Although they do not metastasise, they may cause great local problems which can eventually become lethal or require mutilating surgery. Because palpable lesions are often the 'tip of the iceberg' and because it is difficult to distinguish a clear margin of these tumours on CT or MRI-scan,[1] radical surgery is frequently difficult. Moreover, tumours often recur after surgical excision: even if microscopic margins were clear on pathological examination, local control rates average 72%.[2] Furthermore, and as stated, in pursuing radical surgery important mutilation can be inflicted upon the patient. In view of this, tumour mass reduction by treatment other than surgery is appealing. Weiss and Lackman reported a low-dose chemotherapy regimen involving weekly administration of methotrexate (MTX), at a dose of 50 mg and vinblastine (VBL), at a dose of 10 mg, or MTX 50 mg weekly with oral etoposide 50 mg daily, all for a total treatment duration of 1 year.[3,4] They reported a long-lasting complete or partial remission in 28 of their 36 patients, while only seven patients experienced a transient polyneuropathy as adverse event. Based on these results we routinely applied the above-mentioned regimen of MTX and VBL in patients presenting with primary or recurrent desmoid tumours at sites requiring mutilating surgery. Since we encountered side effects that seem more severe than previously reported, we analysed our data and report on our experience.

### Patients and methods

Patients were eligible for chemotherapy if they met the following criteria: age > 18 years, performance score 0–1 (WHO), histologically proven primary or recurrent desmoid tumour requiring mutilating surgery, white blood cell count $> 4.0 \times 10^9$/l, platelets $> 100 \times 10^9$/l, normal serum creatinine and serum bilirubin, no sign of peripheral neuropathy.

Correspondence to: Dr. J. Verweij, Department of Medical Oncology, Erasmus Medical Center – Daniel den Hoed Cancer Center, Grone Hilldijk 301, 3075 EA Rotterdam, The Netherlands. Tel.: +31-104391911; Fax: +31-104391011; E-mail: verweij@onch.azr.nl

1357-714X print/1369–1643 online © 2003 Taylor & Francis Ltd
DOI: 10.1080/13577140310001644779

Treatment consisted of weekly administration of MTX at a dose of 50 mg and VBL at a dose of 10 mg, both given as a short intravenous bolus infusion. Dose modifications were individualized, depending on the observed side effects. In case of nausea and/or vomiting intravenous administration of 8 mg ondansetron was prophylactically applied on day 1 and oral metoclopramide was prescribed at a daily dose of 20–60 mg. The administration of chemotherapy was scheduled to be given for a total of 52 weeks. Prior to treatment a medical history was taken and physical examination was performed. Full blood counts and serum chemistry (creatinine, sodium, potassium, bilirubine, alkaline phosphatase, AST, ALT, and total protein) were assessed. During treatment, blood counts were taken weekly, biochemistry was assessed every 4 weeks.

Toxicity was assessed weekly and graded according to NCI-CTC criteria, version 2.0. Response was assessed by repeated tumour assessment (usually by means of CT-scan) every 8–12 weeks. WHO response criteria were used.

### Results

Between 01-09-1996 and 31-12-2001, 10 patients with primary or recurrent symptomatic desmoid tumours were treated with chemotherapy. Patient characteristics are shown in Table 1. Four patients had previously undergone surgical resection. Two of them had received multiple treatments: one patient was treated with an isolated limb perfusion with tumour necrosis factor and melphalan after the first resection, followed by re-resection; another patient was treated with radiotherapy after the first resection, followed by re-resection.

Median follow-up was 30 months (10–78 months). The recorded side effects and their consequences are depicted in Table 2. Of note, all patients experienced nausea varying from grade I to III despite anti-emetics. Nausea mainly occurred on days 2 and 3 after the intravenous drug administration. Four patients developed polyneuropathy (grade I–III), four had continuous fatigue (grade I–II), two developed leucopenia (grade II–III) and two patients developed grade II–III impairment of liver functions. The most severe side effect was seen in patient B, who developed dyspnoea grade III during the course of his treatment: a chest X-ray revealed bilateral diffuse interstitial infiltration compatible with MTX-induced interstitial pneumonitis. This diagnosis was histologically confirmed by tissue obtained by open lung biopsy. After termination of the treatment and addition of corticosteroids, the signs and symptoms resolved within a few weeks.

Figure 1 shows the course of the treatment in each patient and their final cumulative dose of MTX and VBL as a percentage of the projected cumulative dose (after 52 administrations). Of note, no patient was able to receive the projected treatment. Six of 10 received less than 50% of the projected cumulative dose, three patients received between 50 and 90% of the dose, while one patient is still on treatment.

One patient achieved a complete remission lasting 21 weeks, seven patients had stable disease. Two of the seven patients with stable disease experienced symptom improvement. Two patients progressed despite their treatment.

### Discussion

In this report we describe an unexpected high level of adverse events in a low dose regimen of MTX and VBL given weekly to patients with desmoids that can only be removed by mutilating surgery. Our experience sharply contrasts with previous reports. While Weiss and Lackman[3,4] reported good tolerance and good activity of this regimen, our results suggest poor tolerance and modest activity. However, as far as activity is concerned we have to be cautious. Our series is much smaller than the one reported by Weiss, and confidence limits for the

**Table 1.** *Patient characteristics*

| Patient | Gender | Age (years) | Tumour site | Primary/ recurrent | Prior treatment | WHO performance score |
|---|---|---|---|---|---|---|
| A | m | 24 | Lower abdominal wall/groin | Primary | – | 0 |
| B | m | 36 | Shoulder girdle | Primary | – | 0 |
| C | m | 45 | Sole of foot | Second Recurrence | Resection, ILP and reresection | 1 |
| D | m | 55 | Infraclavicular thorax | Recurrent | Resection | 1 |
| E | m | 58 | Posterior to clavicle | Primary | – | 1 |
| F | m | 75 | Thoracic wall/axilla | Primary | – | 1 |
| G | F | 17 | Sole of foot | Recurrent | Resection | 1 |
| H | F | 31 | Flexor compartment upper leg | Second Recurrence | Resection, RT and reresection | 1 |
| I | F | 40 | Retro peritoneal | Primary | – | 0 |
| J | F | 44 | Lower pelvis | Primary | – | 1 |

Abbreviations: m, male; f, female; ILP, isolated limb perfusion; RT, radiotherapy.

**Table 2.** *Side effects (worst grade) and treatment consequences*

| Patient | Nausea | PNP | Fatigue | Leucopenia | Impaired LF (AST/ALT) | Other | Comment on chemotherapy |
|---|---|---|---|---|---|---|---|
| A | gr II (10) | | | | gr III (21) | | Treatment delay for MTX (10); subsequent dose reduction for MTX (18); treatment discontinuation (21) all because of impaired LF |
| B | gr I (3) | gr I (3) | | | | Dyspnoea gr III MTX-pneumonitis (22) | Treatment discontinuation (22) because of MTX-pneumonitis |
| C | gr II (3) | | | | | Headache gr I (3) | 50% dose reduction for VBL (3) because of persisting nausea; treatment completion after 1 year |
| D | gr I (12) | | | | | Diarrhoea gr I (12) | Treatment discontinued (18) because of PD |
| E | gr II (4) | gr III * | gr II (7) | | | Alopecea gr I (4) | 50% dose reduction for VBL (7) and finally treatment discontinuation because of persisting nausea, fatigue and PNP* |
| F | gr II (15) | | gr I (5) | gr III (36) | | | Skipping every third dose of VBL (5) and treatment discontinuation (39) because of leucopenia |
| G | gr II (38) | | gr I (12) | | | | Treatment completed after 1 year; delay because of patient compliance |
| H | gr III (3) | gr I (3) | | | gr II (3) | Vomitus gr III (3) | Treatment discontinued (3) because of persisting nausea, vomitus and impaired LF |
| I | gr I (7) | gr I (7) | gr I (7) | | | Diarrhoea gr II (2); Vomitus gr I (7) | 50% dose reduction for VBL (6); deletion of VBL (22) because of diarrhoea and vomitus |
| J | gr II (2) | | | gr II (2) | | Vomitus gr II (14) | 30% dose reduction for MTX; dose interval increase to 10 days (9); treatment discontinuation (14) because of leucopenia and nausea. Also PD |

Abbreviations: PNP, polyneuropathy; LF, liver function; gr, grade of toxicity according to NCI-CTC criteria; PD, progressive disease.
Between parentheses, the number of gifts at which the side effects of worst severity first occurred.
*One-sided neuropathy of the hand, progressively worsening, in a patient with a history of diabetes mellitus and carpal tunnel syndrome.

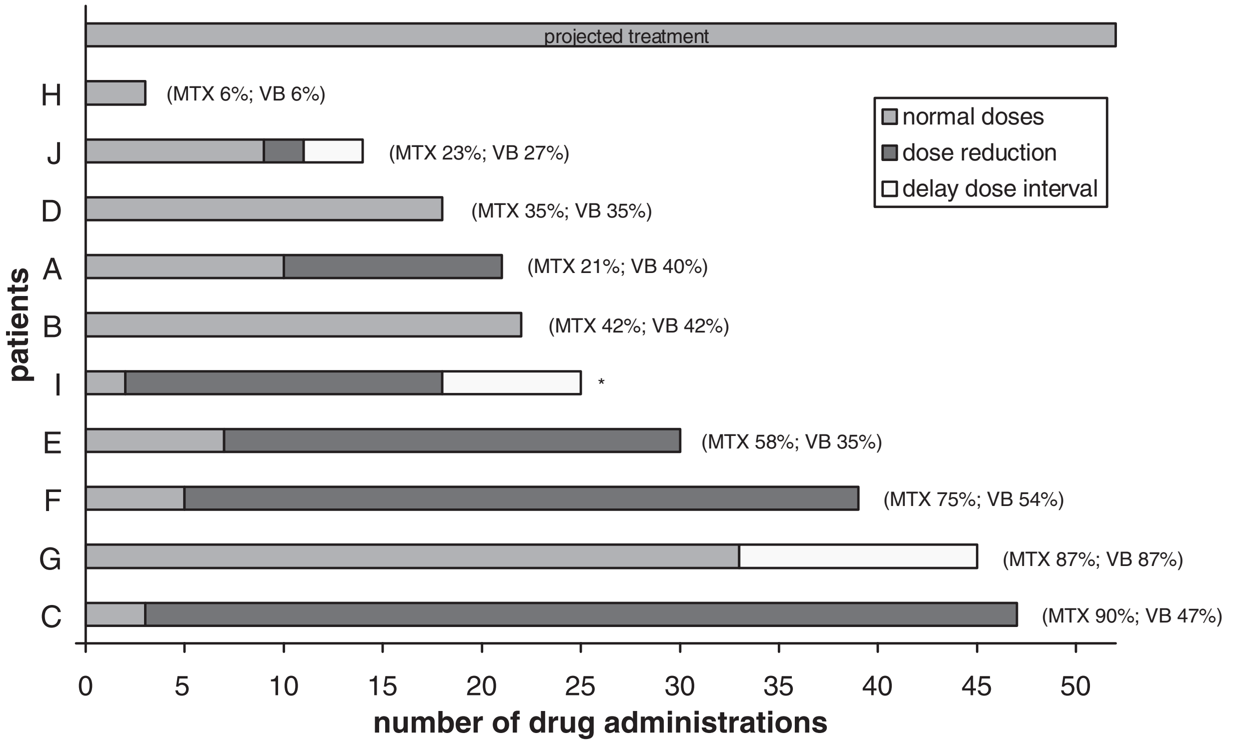

**Fig. 1.** *Projected versus achieved drug administrations and total dose. Between parentheses, the final cumulative dose of MTX and VB given to the patient as a percentage of the projected cumulative dose. *Patient I still on treatment.*

observations are thus large. In addition, it is difficult to assess potential differences in patient selection. We simply may have selected patients with disease characteristics that are less favourable for response to chemotherapy. More of concern is the observed level of toxicity, including severe organ toxicity. The occurrence of a MTX-induced interstitial pneumonitis is rare but well documented.[5] Furthermore, the drug is known to induce hepatotoxicity and we had to stop treatment in two patients due to a progressive relatively severe grade of transaminitis. Fortunately this side effect was reversible. Of further concern is the level of neurotoxicity that we observed. Recently Weiss *et al.*[6] have suggested to replace VBL by vinorelbine, a drug that induces less neurotoxicity but is not devoid of this side effect. Obviously a schedule of MTX and vinorelbine would still bear the risk of MTX toxicity.

Due to the high incidence of various degrees of toxicity, we were unable to administer the projected drug administrations and doses in all patients. Importantly, in six of them it is already evident that we could administer less than 50% of dose and administrations. These findings are in accordance with the results of Azzarelli *et al.*:[7] they undertook a phase II study and treated 30 patients with the same regimen and were only able to treat one patient for a full year and the median dose interval was 10 days. In 15 patients (50%), it was decided to discontinue treatment within 6 months. Four of them received fewer than 15 cycles of chemotherapy, mainly because of severe myelotoxicity. It was acknowledged that the treatment was toxic but that it was useful and that there seemed to be an advantage to attempting to treat beyond 20 weeks, if possible. However, in our series, treatment was delayed or discontinued because of other reasons than myelotoxicity in eight of 10 patients. We think that already in a phase I protocol design such a low level of tolerance would lead to the conclusion that the scheduled dose exceeds the maximum tolerable dose. In any regimen with frequent low dosing, issues such as long-term tolerance and achieved long-term dose intensity should be taken into account. Based on our results, we cannot recommend this dose and schedule of MTX and VBL for routine use.

Desmoids are relatively rare tumours, which is reflected by the low number of publications involving substantial sample sizes. Beyond any doubt, radical resection with clear margins is still the mainstay of treatment.[8] The additive value of radiotherapy to achieve local control has been suggested[2] but, due to the limited size of prospective studies performed, the role of radiotherapy cannot yet be determined with full certainty. This calls for prospective studies on the relevance of treatment interventions and such studies can likely only be performed in cooperative group settings or even intergroup collaborations. Recently the EORTC Soft Tissue and Bone Sarcoma Group initiated a radiotherapy study. The objective of this study will be to determine the efficacy of moderate doses of radiotherapy for desmoid tumours and it will indicate if prospective studies are feasible within a limited time frame.

In conclusion, MTX and VBL at this dose and schedule lead to unacceptable toxicity and impossibility to complete treatment. Other regimens will have to be developed. Performance of prospective trials in cooperative group setting is recommended.

### References

1. Quinn SF, Erickson SJ, Dee PM, Walling A, Hackbarth DA, Knudson GJ, Mosely HS. MR imaging in fibromatosis: results in 26 patients with pathologic correlation. *Am J Roentgenol* 1991; 156: 539–42.
2. Nuyttens JJ, Rust PF, Thomas CR Jr , Turrisi AT III. Surgery versus radiation therapy for patients with aggressive fibromatosis or desmoid tumors. A comparative review of 22 articles. *Cancer* 2000; 88: 1517–23.
3. Weiss AJ, Lackman RD. Low-dose chemotherapy of desmoid tumors. *Cancer* 1989; 64: 1192–4.
4. Weiss AJ, Lackman RD. Long term follow-up of chemotherapy of desmoid tumors and fibromatosis. [Abstract]. *Proc Am Soc Clin Oncol* 1996; 15: 453.
5. Zisman DA, McCune WJ, Tino G, Lynch JP 3rd. Drug-induced pneumonitis: the role of methotrexate. *Sarcoidosis Vasc Diffuse Lung Dis* 2001; 18(3): 243–52.
6. Weiss AJ, Horowitz S, Lackman RD. Therapy of desmoid tumors and fibromatosis using vinorelbine. *Am J Clin Oncol* 1999; 22(2): 193–5.
7. Azzarelli A, Gronchi A, Bertulli R, Tesoro Tess JD, Baratti D, Pennacchioli E, Dileo P, Rasponi A, Ferrari A, Pilotti S, Casali PG. Low-dose chemotherapy with methotrexate and vinblastine for patients with advanced aggressive fibromatosis. *Cancer* 2001; 92: 1259–64.
8. Shields CJ, Winter DC, Kirwan WO, Redmond HP. Desmoid tumours (review). *Eur J Surg Oncol* 2001; 27: 701–6.
